# Supplementary material for: Successional Shifts in Soil C:N:P stoichiometry and its environmental controls during forest restoration in Huize County, Qujing, Yunnan Province
Source: PLoS One. 2026 Aug 21;21(8):e0356261. doi: 10.1371/journal.pone.0356261 (PMC13496410; doi:10.1371/journal.pone.0356261)
Supplement: Supplementary file 2 [file pone.0356261.s004.zip › Data and Code/RF/Pinus yunnanensis forest/figures/importance_C.N.pdf]

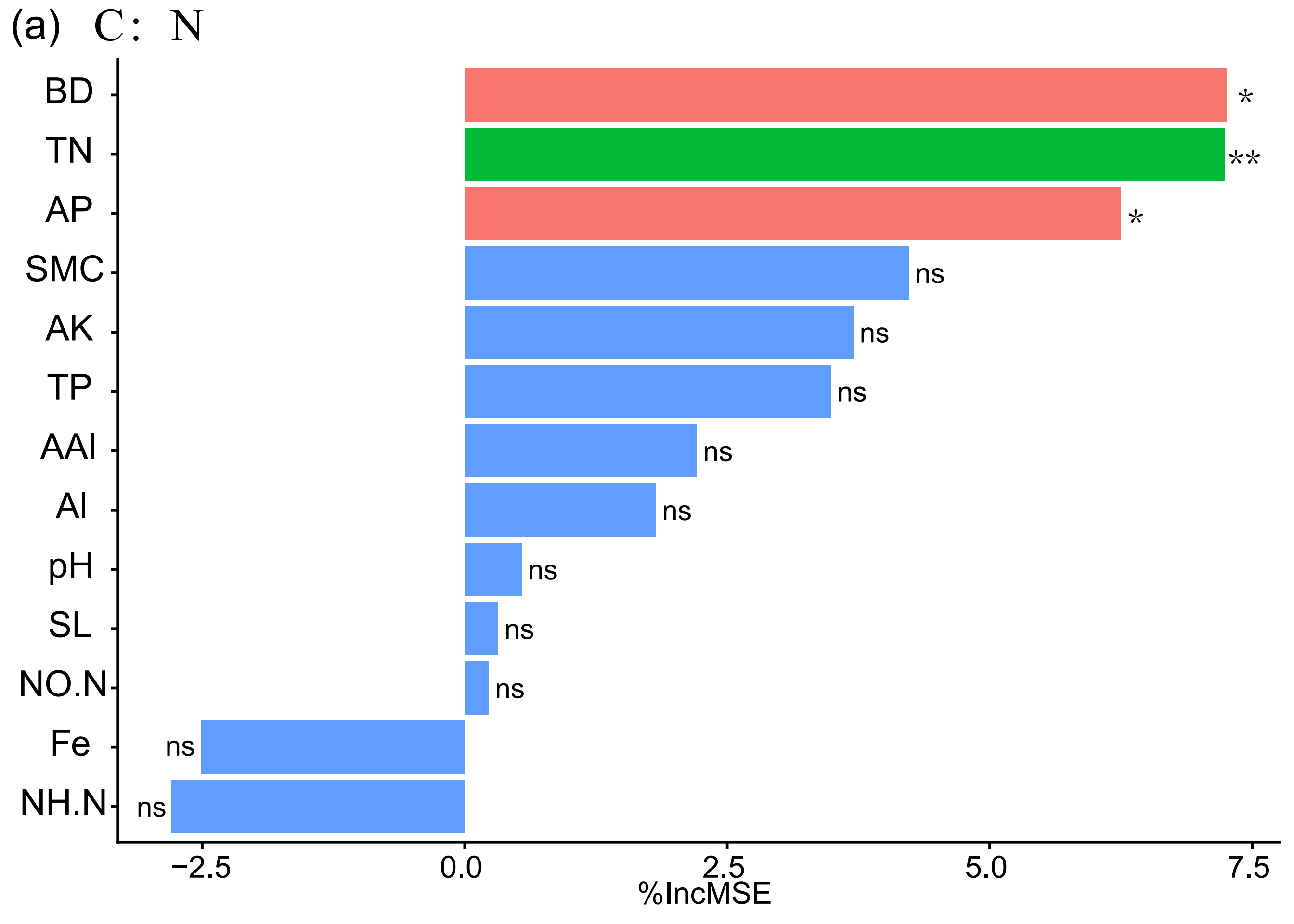

(a) C： N
BD
TN
AP
SMC
AK
TP
AAI
AI
pH
SL
NO.N
Fe
NH.N
*
**
*
ns
ns
ns
ns
ns
ns
ns
ns
ns
ns
−2.5
0.0
2.5
5.0
7.5
%IncMSE
